# Quantification of correlational selection on thermal physiology, thermoregulatory behavior, and energy metabolism in lizards

**DOI:** 10.1002/ece3.1548

**Published:** 2015-08-07

**Authors:** Paulina Artacho, Julia Saravia, Beatriz Decencière Ferrandière, Samuel Perret, Jean-François Le Galliard

**Affiliations:** 1 Instituto de Ciencias Ambientales y Evolutivas, Campus Isla Teja, Universidad Austral de ChileValdivia, Chile; 2 CNRS, UMR 7618, iEES Paris, Université Pierre et Marie Curie7 Quai St. Bernard, 75005, Paris, France; 3 CNRS/ENS UMS 3194, CEREEP – Ecotron IleDeFrance, École Normale Supérieure78 rue du Château, 77140, St-Pierre-lès-Nemours, France

**Keywords:** Ecological energetics, locomotor performances, natural selection, reptiles, thermal coadaptation

## Abstract

Phenotypic selection is widely accepted as the primary cause of adaptive evolution in natural populations, but selection on complex functional properties linking physiology, behavior, and morphology has been rarely quantified. In ectotherms, correlational selection on thermal physiology, thermoregulatory behavior, and energy metabolism is of special interest because of their potential coadaptation. We quantified phenotypic selection on thermal sensitivity of locomotor performance (sprint speed), thermal preferences, and resting metabolic rate in captive populations of an ectothermic vertebrate, the common lizard, *Zootoca vivipara*. No correlational selection between thermal sensitivity of performance, thermoregulatory behavior, and energy metabolism was found. A combination of high body mass and resting metabolic rate was positively correlated with survival and negatively correlated with fecundity. Thus, different mechanisms underlie selection on metabolism in lizards with small body mass than in lizards with high body mass. In addition, lizards that selected the near average preferred body temperature grew faster that their congeners. This is one of the few studies that quantifies significant correlational selection on a proxy of energy expenditure and stabilizing selection on thermoregulatory behavior.

## Introduction

How phenotypic selection (variation in fitness associated with one or a combination of phenotypes) shapes the adaptation of complex set of morphological, physiological, and behavioral traits is one of the most intriguing questions in evolutionary biology (Feder et al. 2000; Irschick et al. 2008; Kingsolver et al. 2012). Fitness is rarely determined by a single trait; instead, many traits, including morphology, behavior, and functional properties, interactively affect fitness and should therefore evolve in concert (e.g., Arnold 1983; Svensson et al. 2001; reviewed in Sinervo and Svensson 2002). In ectotherms, the coevolution of the thermal physiology of locomotor performances, thermoregulatory behavior, and energy metabolism is of special interest because of their potential coadaptation (Huey and Bennett 1987; Huey and Kingsolver 1989; Garland et al. 1991; Angilletta et al. 2006). A central tenet of thermal biology is that individuals should select body temperatures that optimize their physiological performances, such that thermoregulatory behavior and thermal physiology are coadapted (reviewed in Angilletta 2009). Energy metabolism is also intimately linked to the thermal biology and performance. Although most ectotherms do not use metabolism to regulate their body temperature, behavior, performance traits, and maintenance metabolism could contribute simultaneously to growth, reproduction, and survival (Careau and Garland 2012; Artacho et al. 2013).

In squamate reptiles (lizards and snakes), empirical studies of thermal coadaptation have focused on interspecific comparisons of preferred body temperatures (PBT), thermal sensitivity of locomotor performances, and the maintenance cost represented by the resting metabolic rate (Angilletta et al. 2002; Careau and Garland 2012; Huey et al. 2012). Those traits are defined, respectively, as the mean selected body temperature in a thermal gradient free of the costs of thermoregulation, the thermal performance curve of a locomotor capacity such as maximal sprint speed (Huey and Stevenson 1979), and the lowest metabolic rate of an individual recorded at a rest under less restrictive conditions than the basal metabolic rate (McNab 1997). A locomotor performance curve typically increases with temperature up to an optimum after which performance drops down rapidly until a critical, lethal thermal maximum is reached. Research about thermal coadaptation has discussed whether the performance breadth and the maximal performance trade off and whether a warm-adapted genotype has a higher maximal performance (“hotter is better” hypothesis, Huey and Kingsolver 1989; Angilletta 2009). However, interindividual variation in the thermal optimum and the performance breadth is usually difficult to assess because it requires measurements close to lethal conditions, accurate estimates around the optimum, and appropriate nonlinear models of the performance curve (Angilletta 2006). In order to quantify selection on relevant thermal biology traits, we focused instead here on the thermal sensitivity, defined as the rate of increase of locomotor performance across a natural range from low to optimum body temperature. We predict that behavioral thermoregulation at higher body temperatures together with a higher thermal sensitivity of locomotor performances may be advantageous for fitness if not traded off against potential costs of thermoregulation and lower scores of other physiological performances. Thus, we should find a positive correlational selection gradient on the PBT and the thermal sensitivity of the locomotor performance traits (see Table1, “hotter is better” scenario).

**Table 1:** Main evolutionary scenarios of thermal coadaptation and general predictions about natural selection gradients in ectotherms. The “hotter is better” model was reviewed by Angilletta et al. (2002) and models of ecological energetics were reviewed by Careau and Garland (2012).

Evolutionary scenario	Main predictions about natural selection gradients in ectotherms
“Hotter is better model” of thermal biology	Directional selection for behavioral thermoregulation at higher PBT
	Directional selection for higher thermal sensitivity of locomotion
	Positive correlational selection on PBT and thermal sensitivity
“Allocation model” of ecological energetics	Negative directional selection on RMR (maintenance)
	Positive directional selection on locomotor activity and PBT (production)
	Negative correlational selection between RMR and production (locomotion and PBT)
“Production model” of ecological energetics	Positive directional selection on RMR through production
	Positive directional selection on locomotor activity and PBT
	Positive correlational selection between each pair of the three traits
	Potential survival costs of higher production (e.g., higher predation)

PBT, preferred body temperature; RMR, resting metabolic rate.

In addition, there is growing evidence that metabolism can have an effect on fitness through its influence on energy allocation, including maintenance, activity, or reproduction (Artacho and Nespolo 2009; Larivee et al. 2010; Burton et al. 2011; Careau and Garland 2012). In ectotherms, the resting metabolic rate (RMR) corrected for body mass increases on average with body temperature and represents a significant component of the total energy budget that is intimately linked with the cost of life (e.g., Artacho and Nespolo 2009). Low resting metabolic rates might confer an advantage in fitness by decreasing the cost of life because of trade-offs between maintenance, growth, and reproduction (e.g., Steyermark 2002). Thus, lower RMR, in combination with the behavioral selection of higher body temperatures and with a higher locomotor activity, should increase simultaneously individual fitness (see Table1, “allocation model” scenario). Alternatively, RMR could be considered as a by-product of other enhanced whole-organism physiological traits that increase production (Burton et al. 2011). For example, high resting metabolic rates could be associated with higher metabolic scope, higher food intake, and digestion capacity. In this case, augmenting the combination of RMR, PBT, and one locomotor performance trait would be expected to increase growth and reproduction, possibly at the cost of lower survival (see Table1, “production model” scenario). In addition, fitness differences between these traits may be influenced by the body mass, because body mass is positively correlated with metabolism and has an indirect link with fitness through performance traits. Yet, no study has examined how phenotypic selection within natural populations can simultaneously act on thermal and energetic traits (Angilletta et al. 2002; Anderson et al. 2011; Logan et al. 2014). Thus, the question of whether these traits determine independently and additively fitness or interact to determine fitness, like suggested by the concept of coadaptation, remains open.

Here, we quantified the strength and direction of phenotypic selection on morphology (body mass), thermoregulatory behavior (preferred body temperature), thermal sensitivity of locomotor performance (rate of increase of maximal sprint speed with body temperature), and resting metabolic rate corrected for body mass in ten independent, seminatural populations of common lizards (*Zootoca vivipara*) maintained in similar environmental conditions. Maximal sprint speed is a relevant locomotor performance in lizards because it is involved in foraging, predator escape, and territorial contests, and is positively correlated with survival in several species (Irschick et al. 2008). In a previous study, we found repeatable interindividual variation for all traits in a large sample of subadult and adult common lizards, as well as nonsignificant phenotypic correlations among them (Artacho et al. 2013). In this study, lizards measured for all traits in the laboratory were released in outdoor enclosures during the middle of the summer (active season) and were recaptured during the following spring (breeding season). This allowed to examine three components of annual fitness (survival, growth, and fecundity) in seminatural conditions in the presence of competition among all age and sex classes of common lizards and of avian predation, which are important ecological determinants of natural selection in this species (Le Galliard et al. 2004, 2015). In lizards, correlational selection on thermal biology and energy metabolism has never been quantified (Le Galliard et al. 2013), and our data thus make it possible to test predictions about the shape of natural selection for the first time (Table1).

## Materials and Methods

### Model species and maintenance conditions

The common lizard, *Zootoca vivipara*, is a small diurnal and insectivorous lacertid (50–70 mm adult snout-vent length) living in peat bogs and heath land across Eurasia. Two hundred and four individuals (females and males of 1-year-old, 2-year-old, and more than 2-year-old age classes) were captured by hand during June 2010 in enclosed seminatural populations located in a meadow at the Centre de Recherche en Ecologie Expérimentale et Prédictive, France (48° 17′ N, 2° 41′ E). All animals had been marked at birth with a unique code by mean of toe clipping. After capture, animals were maintained under standard conditions in individual boxes (10/14 day/night cycle, 15°C at night, 23–35°C thermal gradient at day, permanent access to water) and fed ad libitum every second day with crickets (*Acheta domestica*). All traits were measured in the animals during the postbreeding period (July 26 to August 18) following laboratory protocols described in details in Artacho et al. (2013).

### Measurements of preferred body temperature

Selected body temperatures were measured under constant artificial white light (Reptisun 10.0 UVB, ZooMed) in a thermal gradient (77 cm long) ranging from room temperature (21–22°C) at an end to 40–42°C under an incandescent bulb (40 W) at the other end. Lizards were placed in the gradient the evening before measurements to allow acclimation, and body temperature was measured with an infrared thermometer every 30 min from 10:30 to 17:00 local time. Readings were considered estimates of behaviorally selected body temperatures. In addition, the location of each lizard in the terrarium was classified as near the heat source and above the substrate, near the heat source and hidden in the soil, far the heat source and above the substrate, and far the heat source and hidden in the soil. Preferred body temperature (PBT) was calculated as the daily average of selected body temperatures. We excluded the values recorded at the latter position (352 records of 2647) because they represent lizards at rest rather than in active thermoregulation and were more frequently observed at the beginning and at the end of the day, when lizards may not be active (see Artacho et al. 2013). Preferred body temperature was positively correlated with thermal precision, implying that lizards selecting higher temperatures were also more accurate thermoregulators.

### Measurements of thermal sensitivity of maximal sprint speed

We characterized sprint speed at five body temperatures (20°, 24°, 28°, 32°, and 36°C) normally reached by common lizards during the activity season. We used a 2.5 m long linear racetrack covered with a cork floor and equipped with photoelectric cells spaced by 25 cm. Each lizard was placed on one end of the racetrack and chased by gently tapping the tail with a soft brush (quantified as a stimulation index). Individuals were randomly assigned to groups of approximately 12 individuals, and the sequence of temperatures for each group was also established in a random fashion. Each lizard was run at all temperatures on two consecutive days, and body temperature was manipulated by keeping individuals in an environmental chamber for at least 1 h. The fastest speed of three trials estimated over a 25-cm interval was considered the estimate of maximal sprint speed (MSS, cm·sec^−1^). We then estimated the best linear unbiased predictors of the intercept and slope of the relationship between maximal sprint speed and body temperature for each individual by mean of a mixed-effects model with a restricted maximum-likelihood approach (Pinheiro and Bates 2000). The best final model included the linear and quadratic effect of body temperature, sex, age, and stimulation index as fixed factors, and the slope and intercept of regression per individual between MSS and body temperature as random effects (Artacho et al. 2013). Body size was not influential implying that MSS was not confounded by morphology. For the selection analysis presented here, we used the random slope to estimate the thermal sensitivity of sprint speed of each individual (Nussey et al. 2007). However, the random slope was positively correlated with random intercept (*r*^2^ *= *0.707, *P < *0.001). Thus, animals that had higher thermal sensitivity also had higher mean speeds and, also probably, maximal performances at the optimal body temperature. In this case, a nonsignificant effect of thermal sensitivity on fitness would apply to mean, and possibly maxima, sprint speed performances as well.

### Measurements of resting metabolic rates

Resting metabolic rate was estimated in fasting lizards at rest during the daytime through an open-flow respirometric system (Qubit Systems, Kingston, ON, Canada). Briefly, metabolic rates were estimated with a system compounded of a differential oxygen analyzer (DOX, S104 Differential Oxygen Analyzer) and a CO_2_ analyzer (S157) connected to a respirometry software (QS Research). Incoming air flowed through columns of soda lime and Drierite to remove CO_2_ and H_2_O, respectively, and it was pushed at 140 mL·min^−1^. To ensure the postabsorptive state of the lizards, metabolic rate was measured at 20°C after a period of fasting of 72 h. Lizards were allowed to acclimate to chambers during 1 h, and we measured outgoing CO_2_ and O_2_ concentrations every sec. during half an hour. Individuals were weighted after removal from the chambers. Baselines of CO_2_ and O_2_ concentrations were recorded each 1 h of recording. Respiratory quotient (RQ) was used to convert CO_2_ production values (mL·h^−1^) to energy expenditure (J per hour, Walsberg and Wolf 1995). We took the average of each complete record as the estimation of resting metabolic rate. As RMR was weakly correlated with body mass (Artacho et al. 2013), we used residual RMR (residual of a log–log regression between RMR and body mass) as a covariate in our statistical analyses.

### Captive population study

After completion of all measurements, animals were released and maintained over a period of 9 months (i.e., between end of August 2010 and end of May 2011) in 10 separate enclosures of 12 × 8 m in a natural meadow located at CEREEP, France. This time period embraces late summer activity season, winter hibernation, winter emergence, and spring mating period. Enclosures were surrounded by plastic walls to prevent the escape of lizards, but were not protected from avian predators (crows, magpies, and kestrels, pers. obs.) and provided lizards with natural access to food and water (no additional food was provided). Each enclosure had approximately the same statistical distribution of body mass, sex, and age classes of lizards (see Table S2). In addition, we released at random 35 newborns in each enclosure to match the stable age structure and density of wild populations (Le Galliard et al. 2004). This procedure ensured that environmental conditions experienced by lizards were homogeneous, allowed for strong food and space competition and avian predation, and made it possible to quantify selection gradients while minimizing heterogeneity due to migration out of o study area and/or capture probability. Mortality, growth, and reproductive traits in these enclosures are within the range of variation seen in nature (Mugabo et al. 2013).

At the end of May 2011, we walked inside the enclosures several hours per day during 2 weeks and captured by hand all surviving lizards. Lizards that were not recaptured during this census were considered dead. Surviving animals were carried out to the laboratory for the measurement of snout-vent length (hereafter, SVL). Body growth was measured as the difference in SVL between after and before the selection study. In addition, females were maintained in individual boxes under standard conditions (same as above) until they produced their single litter of the year (during the two last weeks of June). We counted the total fecundity, including the total number of unhatched eggs, dead hatchlings, and live hatchlings, but obtained similar results when we focused our analyses on total number of live hatchlings.

### Quantification of selection gradients

All selection analyses were carried out using the software R, version 2.10 (R Development Core Team, 2014). We used multivariate regressions to quantify linear, quadratic, and correlational selection gradients for survival, growth, and reproduction after all traits were standardized to zero mean and unit variance (Lande and Arnold 1983; Brodie and Janzen 1996). We followed advices of Stinchcombe et al. (2008) to calculate selection gradients. Enclosure identity was included in all models as a random effect to control for nonindependence among observations from the same enclosure. We measured selection via survival (*N* = 204) assigning 1 for surviving lizards and 0 for dead lizards (i.e., individuals were not recaptured) and used a logistic regression to test the significance of the selection gradients (Janzen and Stern 1998). We started the analysis by making a full model that included the following variables: body mass, PBT, residual RMR, thermal sensitivity of the MSS (represented by the slope, see above), the quadratic term of each variable, the cross-product between each pair of variables, and the factors sex and age. This model fitted well the data according to a goodness-of-fit test. A minimum adequate model was then selected with a stepwise procedure by likelihood ratio tests (LRT) in the *lmer* procedure. Associated P values of LRT and confidence intervals of estimates were obtained by parametric bootstraps following Faraway^2013^ (2013, with 1000 bootstrap replicates).

We followed a similar approach for the analysis of body growth (*N* = 115) using a linear regression with the *lme* procedure and LRT tests based on the maximum-likelihood approach (Pinheiro and Bates 2000). Assumptions of normality and homogeneity of variance were fulfilled for this analysis. We further analyzed selection via fecundity in the subset of surviving females (*N* = 61). We tested for the effects of PBT, residual RMR, and thermal sensitivity of the MSS on the total annual fecundity using a linear regression with the *lme* procedure. Because the sample size of reproductive females was small, we used a forward selection approach to select the minimum adequate model. We started from the simple intercept model and tested for all main effects, all quadratic effects, and all two-way interactions in a sequential manner until no significant terms could be added to the model. Phenotypic traits were weakly correlated (Artacho et al. 2013), but we were unable to measure all traits in all individuals (see Table S1 for sample size and mean values). Thus, sample size varied between models when some covariates were eliminated during the backward selection. Of the 204 individuals released inside the 10 enclosures, we were able to recapture 115 live animals. The survival in one enclosure was low (only four individuals while numbers ranged between nine and 14 survivors in other enclosures). This lower survival could be due to overpredation, unknown interindividual differences, or random mortality events. For that reason, we tested for robustness of the results when this enclosure was removed or not from the data set. We removed these observations in the main analyses but also report the results for all data as well because they were similar. Marginal (including fixed effects only) and conditional (including fixed and random effects) pseudo-*R*^2^ and difference in AIC_c_ with the null model were calculated following Nakagawa and Schielzeth (2012).

## Results

**Figure 1:**
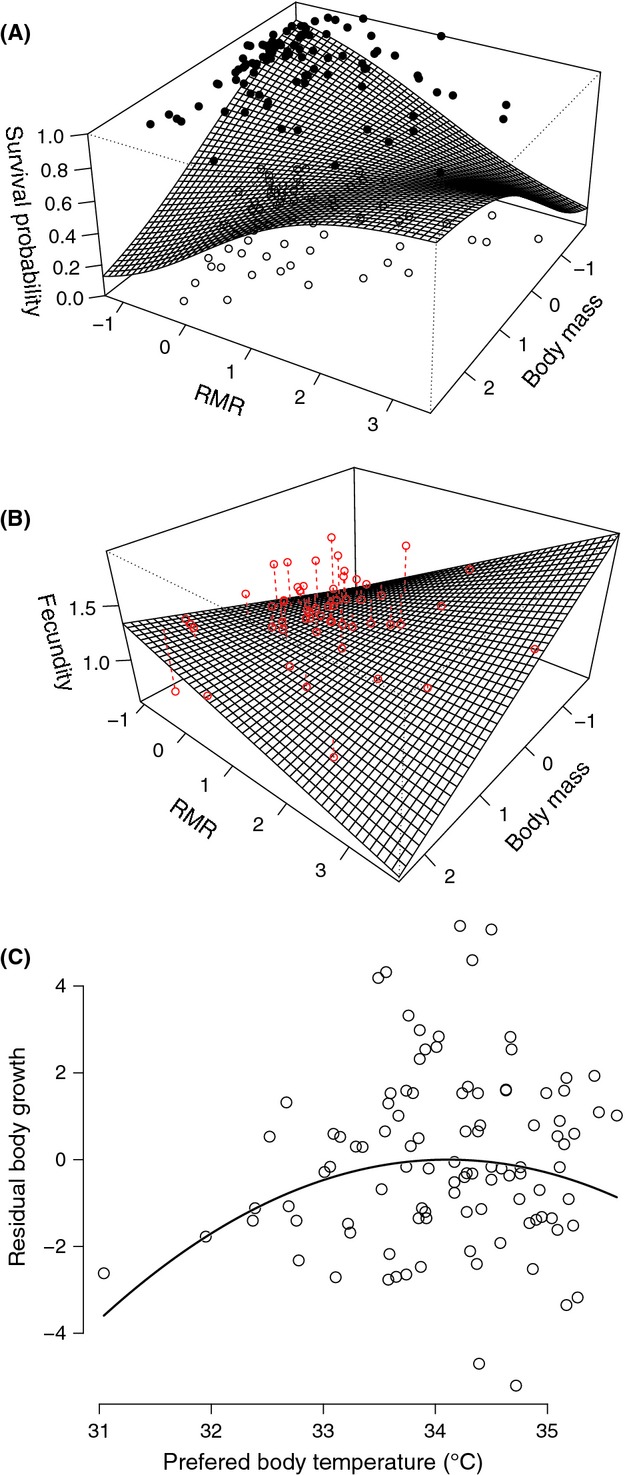
Predicted fitness curves for annual survival, total fecundity, and body growth in *Zootoca vivipara*. The annual survival (A) and total fecundity (B) were significantly influenced by correlational selection acting on body mass and RMR (corrected for body mass). Results are raw data and predicted fitness curves obtained from the best models in Table2 and Table3, respectively. In (A), filled circles indicate survivors, while empty circles indicate dead animals. In (B), dotted lines from observed to predicted values were also drawn. Body growth (C) was influenced by a weak stabilizing selection on preferred body temperature. Results are residual values of body growth after accounting for the effects of sex and age (see main text for the best model). The apparent increase in residual variability with PBT was close to significance (heteroscedasticity modeled with a power variance structure, LRT test = 3.70, *P* = 0.05). Accounting for this heteroscedasticity did not change the significance of the quadratic effect of PBT (*F*_1,89_ = 5.69, *P = *0.036).

Neither age, sex, PBT, nor thermal sensitivity of MSS significantly influenced annual survival (all *P* > 0.05), but we quantified significant correlational selection between body mass and residual RMR (see Table2, bootstrap LRT test: *P* = 0.02 for the interaction term; all data: *P* = 0.044). Thus, viability selection favored lizards with high body mass in concert with high energy maintenance costs represented by high residual RMR and favored also lizards with low body mass and low energy maintenance costs (Table2, Fig.1A). In between these two fitness peaks, there was a valley for a group of individuals characterized by a negative correlation between body mass and residual RMR.

**Table 2:** Quantification of correlational viability selection on body mass and resting metabolic rate. Data are from the best selected mixed-effects logistic regression designed to quantify viability selection. Traits were standardized to mean 0 and standard deviation 1 prior to analysis. Standardized selection gradients were calculated according to the methods of Janzen and Stern (1998). The model explained little variation in the data (marginal *R*^2^ = 0.07, conditional *R*^2^ = 0.11, ΔAIC_c_ = 26.45 with df = 3).

	Model estimate± SE	*Z* value	*P* value	Selection gradient
Fixed effects				
Intercept	0.406 ± 0.162	2.472	0.013	–
Body mass (g)	−0.271 ± 0.166	−1.635	0.102	*β* = −0.103
Residual RMR (J·h^−1^)	−0.040 ± 0.168	−0.197	0.844	*β* = −0.015
Body mass × RMR	0.422 ± 0.191	2.216	0.027	*γ* = 0.160

Residual RMR, resting metabolic rate corrected for body mass.

The average of total fecundity was 6.36 (±1.9 SD, range = 3–11). Like for survival, we did not find effects of age, PBT, or thermal sensitivity of MSS on total fecundity (all *P* > 0.05), but we quantified significant correlational selection on body mass and residual RMR (Table3; all data, interaction term: *F*_1,43_ *=* 8.04, *P* = 0.007). In this case, correlational selection implied that total fecundity was higher along a ridge corresponding to a group of individuals with a negative correlation between body mass and residual RMR, while a lower fitness was seen for females with both high body mass and high residual RMR, and for females with both low body mass and low residual RMR (Fig.1B).

**Table 3:** Quantification of correlational fecundity selection on body mass and resting metabolic rate. Data are from the best linear mixed-effects model used to quantify variation in the standardized fecundity of surviving females (i.e., total fecundity divided by the mean of the sample). Traits were standardized to mean 0 and standard deviation 1 prior to analysis. The model explained well variation in the data (marginal *R*^2^ = 0.24, conditional *R*^2^ = 0.25, ΔAIC_c_ = 7.42 with df = 3).

	Selection gradient ± SE	*F*-test_[1,43]_	*P*-value
Fixed effects			
Intercept	1.017 ± 0.037	–	–
Body mass (g)	0.067 ± 0.036	3.405	0.072
Residual RMR (J·h^−1^)	0.099 ± 0.037	7.232	0.010
Body mass × Residual RMR	−0.104 ± 0.037	7.642	0.008
Random effects	Estimate (95% CI) of standard deviation		
Enclosure identity	0.029 (0.00007, 11.930)		
Residuals (within enclosures)	0.249 (0.204, 0.305)		

RMR, resting metabolic rate corrected for body mass.

Body growth was higher in females than in males (*F*_1,89_ = 112.67, *P < *0.001; contrast = +4.41 mm ±0.41 SE) and in 1-year-old individuals compared with the older individuals (*F*_2,89_ = 54.1, *P *< 0.001), but growth was not influenced by body mass, residual RMR, and thermal sensitivity of MSS (all *P* > 0.05). In addition, we found stabilizing selection on PBT, such that body growth was maximized when selected body temperatures were close the population mean (quadratic effect of PBT: *F*_1,89_ = 4.55, *P = *0.036, quadratic selection gradient: −0.516 ±0.28 SE; analysis with all data: *F*_1,92_ = 4.31, *P = *0.04; see Fig.1C). This model explained well variation in body growth rate (marginal *R*^2^ = 0.68, conditional *R*^2^ = 0.71, ΔAIC_c_ = 110.55 with df = 4).

## Discussion

We analyzed selection on two thermal biology traits, energy metabolism and body mass, in a large sample of lizards in outdoor enclosures. We quantified multivariate selection gradients during 1 year, which is useful to understand the shape and strength of selection and to predict the responses to selection of multiple traits (Lande and Arnold 1983; Brodie and Janzen 1996; Stinchcombe et al. 2008). However, potential pitfalls with this approach include the possibility of statistical biases in estimates of nonlinear selection, temporal variability in selection gradients, and difficulties to infer direct and indirect responses in the absence of data about the heritability and genetic covariance of traits (Kingsolver et al. 2012). In addition, as we did not manipulate food availability or thermal conditions inside enclosures, it remains difficult to identify which aspects of the environment are causing the hypothesized selection. Below, we discuss our main findings noting that our quantification of selection gradients should be complemented with further manipulative and quantitative genetic studies.

Our study period embraced two seasonal stages (prehibernation and emergence of hibernation) that are energetically challenging in the yearly cycle of the common lizard because food availability is low, thermal conditions are suboptimal, and behavioral activity is still significant. In addition, adults *Z. vivipara* start to breed a few weeks after winter emergence, which increases the reliance on energetic reserves accumulated the previous summer season to sustain activities during the mating season (Voituron et al. 2000; Bleu et al. 2013). Given this, our most interesting result was the significant correlational selection acting on the residual resting metabolic rate (RMR, corrected for body mass) and body mass in opposite directions for survival and reproduction, such that total selection on the two traits was not divergent. In sharp contrast, we found weak selection gradients on interindividual differences in thermoregulatory behavior and thermal physiology, except for the stabilizing selection gradient on PBT.

Natural selection acting on the minimal cost of maintenance has been interpreted from two opposite points of view called the “production model” and the “allocation model” of ecological energetics (Careau et al. 2008; Biro and Stamps 2010; see Table1). However, there exist only a handful of reports of selection gradients on the RMR, and none evidenced significant correlational selection on RMR and body mass like our study did. In endothermic mammals, directional, positive selection gradient on residual RMR was found in meadow voles *Microtus agrestis* (Jackson et al. 2001) and in bank voles *Myodes glareolus* (Boratynski and Koteja 2010; Boratynski et al. 2010). In free ranging chipmunks *Tamias striatus*, residual RMR was positively correlated with body growth and survival was maximal for individuals with intermediate values of metabolic expenditure (Careau et al. 2013). These results are best interpreted by the production model as RMR is on average positively correlated with fitness. On the contrary, North American red squirrels with low residual RMR and a large body mass, by separate, survived better probably because they minimized expenditure costs and maximized thermal inertia during the winter (Larivee et al. 2010). This is similar to data from two previous studies of ectothermic animals where RMR represents a significant proportion of the total energetic expenditure (juvenile fishes and land snails, Bochdansky et al. 2005; Artacho and Nespolo 2009).

Contrary to these two earlier studies on ectotherms, a combination between high body mass and high resting metabolic rate was positively correlated with survival and negatively correlated with fecundity in the common lizard. The finding that fitness gradients on RMR changed from positive to negative values depending on body mass indicates that different mechanisms underlie selection on metabolism in lizards with small body mass than in lizards with high body mass. In common lizards, differences in body mass are correlated with age (controlled for in this analysis) and early size growth as well as with differences in fat stores, the main energetic reserves. Lizards of a small body mass feed less but invest more in body growth and have less body reserves than lizards of a higher body mass (González-Suárez et al. 2011). In these lizards, a higher residual RMR may represent a more productive lifestyle and consequently higher future fecundity at the expanse of current survival (see Table1, “production model”). This is because high resting metabolic rate cannot be compensated by a higher rate of food intake and/or a stronger reliance on stored energetic resources.

In larger lizards, the stronger reliance on fat store to cover the energetic costs of maintenance implies that survival costs of a more productive lifestyle should be less sensitive to RMR. Thus, viability selection on RMR should flatten but fertility selection might remain positive. Yet, we found evidence of positive viability selection and slight negative fecundity selection on RMR in the largest lizards. One explanation is that the higher sustained energy output conferred by a higher RMR is invested into social dominance and aggressive behaviors to promote survival rather than reproduction in large, grown up lizards (Biro and Stamps 2010). Another possibility is that proximate determinants of individual differences in RMR differ between small and large individuals. In particular, measures of RMR in small, growing individuals include the energy costs of tissue synthesis (i.e., structural growth), which is not the case for fully grown individuals (e.g., Hou et al. 2008). We found no evidence that RMR was correlated with body growth here, but more studies are needed to understand the proximate causes of interindividual differences in resting energy metabolism. In addition, it is difficult to know whether reproduction and body growth was limited by food supply rather than food processing capacity, which may be positively correlated with RMR. Future studies could try to manipulate food supply to see whether food limitation changes selection on RMR as seen in laboratory and field experiments with fishes (Burton et al. 2011).

Contrary to predictions from the “hotter is better” model of thermal biology (Table1), there was no significant selection gradient on thermal sensitivity of maximal sprint speed and no significant correlational selection gradient on thermal physiology and thermoregulatory behavior. In addition, selection on metabolism was independent from thermal physiology and thermoregulatory behavior. Thus, maximal locomotor performances differed among individuals but maximal sprint speed was unrelated to survival, growth, and reproduction. Other studies have also failed to detect significant selection on locomotor performances in lizards (Irschick et al. 2008; Le Galliard and Ferrière 2008), which may be due to limited use of maximal sprint speed in the field by some individuals, ontogenic changes in locomotor performances, or trade-offs between speed and endurance (Irschick and Garland 2001; Le Galliard et al. 2004). In a rare investigation of climate-driven selection on thermal performances of lizards, natural selection was weak in the native range but much stronger in a non-native range characterized by warmer and more variable weather conditions (Logan et al. 2014). This suggests that natural selection on the thermal performance curves may be easier to detect under some circumstances such as during years of extreme climate conditions and in populations located at the margins of the species range.

To our knowledge, our study is, however, the first to report a quantification of the correlational selection gradients between thermal physiology and thermoregulatory behavior. According to thermal coadaptation models (Table1), we would expect a positive correlational selection between preferred body temperature and thermal sensitivity of maximal sprint. Unfortunately, our data did not support this major prediction. Yet, the preferred body temperature (PBT) was under the action of weak stabilizing selection because individuals that showed a PBT near the population mean grew faster than their conspecifics. PBT is routinely measured in reptiles with an active thermoregulation strategy assuming that it represents the body temperature at which several physiological processes (e.g., digestion, locomotion, and gestation) are optimized (Herczeg et al. 2006). In addition, models for the evolution of optimal thermoregulation assume that the preferred body temperature represents a balance between costs and benefits (Huey and Kingsolver 1989; Kingsolver 2009). Support for the cost-benefit model of thermoregulation comes from studies of adaptive phenotypic plasticity in basking behavior, for example, changes in PBT across seasons, reproductive stages, or populations. Our study is the first to report the analysis of selection on interindividual differences in basking behavior within the same population and suggests that the mean PBT is near a local optimum for body growth in this species. Given that the repeatability of preferred body temperature of males of *Z. vivipara* in a 1-month time period was high and significant (Artacho et al. 2013), this trait has thus the potential to respond to the selection.

## Supporting Information

Table S1. Descriptive statistics (mean ± SD) for morphological traits (snout-vent length [SVL] and body mass), preferred body temperature (PBT, °C), resting metabolic rate (RMR, J·h^−1^) and thermal sensitivity (TS, a measure of the maximal sprint speed sensitivity to body temperature corrected for age, sex and behavioral effects) in common lizards (*Zootoca vivipara*). **Table S2.** Number of female (F) and male (M) common lizards released in each experimental enclosure.
