# Supplementary material for: Quantification of correlational selection on thermal physiology, thermoregulatory behavior, and energy metabolism in lizards
Source: Ecol Evol. 2015 Aug 7;5(17):3600–9. doi: 10.1002/ece3.1548 (PMC4567864; doi:10.1002/ece3.1548)
Supplement: Table S1. — Descriptive statistics (mean ± SD) for morphological traits (snout-vent length [SVL] and body mass), preferred body temperature (PBT, °C), resting metabolic rate (RMR, J·h−1) and thermal sensitivity (TS, a measure of the maximal sprint speed sensitivity to body temperature corrected for age, sex and behavioral effects) in common lizards (Zootoca vivipara). Table S2. Number of female (F) and male (M) common lizards released in each experimental enclosure. [file ece30005-3600-sd1.docx]

## Supplementary Tables

**Table S1.** Descriptive statistics (mean ± SD) for morphological traits (snout-vent length [SVL] and body mass), preferred body temperature (PBT, °C), resting metabolic rate (RMR, J.h^-1^) and thermal sensitivity (TS, a measure of the maximal sprint speed sensitivity to body temperature corrected for age, sex and behavioral effects) in common lizards (*Zootoca vivipara*).

|  | One-year old | | Two-years old | | More than two years-old | | Sample size |
| --- | --- | --- | --- | --- | --- | --- | --- |
| Variables | Females | Males | Females | Males | Females | Males |  |
| SVL | 56.58 ± 2.84 | 53.60 ± 1.93 | 65.87 ± 2.85 | 58.86 ± 3.23 | 67.89 ± 2.86 | 59.60 ± 1.53 | 204 |
| Body mass | 3.20 ± 0.37 | 3.19 ± 0.35 | 4.12 ± 0.52 | 3.86 ± 0.55 | 4.63 ± 0.49 | 4.22 ± 0.36 | 204 |
| PBT | 33.87 ± 0.90 | 34.50 ± 0.56 | 34.04 ± 0.78 | 34.54 ± 0.62 | 33.89 ± 0.96 | 33.79 ± 0.97 | 194 |
| RMR | 9.80 ± 6.00 | 10.26 ± 6.71 | 14.61 ± 7.43 | 9.65 ± 3.93 | 11.85 ± 6.56 | 13.09 ± 7.01 | 184 |
| TS | 0.025 ±0.005 | 0.026 ± 0.005 | 0.024 ± 0.004 | 0.027 ± 0.005 | 0.026 ± 0.006 | 0.028 ± 0.006 | 171 |

**Table S2.** Number of female (F) and male (M) common lizards released in each experimental enclosure.

| **Enclosure** | **One-year old** | **Two-years old** | **More than 2 years old** |
| --- | --- | --- | --- |
| 1 | F: 6, M:6 | F:2, M:3 | F:2, M:1 |
| 2 | F:2, M:3 | F:8, M:6 | F:2, M:0 |
| 3 | F:7, M:6 | F:1, M:2 | F:4, M:1 |
| 4 | F:6, M6 | F:2, M:1 | F:3, M:3 |
| 5 | F:7, M:6 | F:1, M:2 | F:3, M:1 |
| 6 | F:8, M:6 | F:1, M:0 | F:3, M:3 |
| 7 | F:7, M:6 | F:2, M:2 | F:2, M:1 |
| 8 | F:7, M:6 | F:1, M:0 | F:3, M:3 |
| 9 | F: 7, M:6 | F:2, M:1 | F:2, M:2 |
| 10 | F:7, M:6 | F:1, M:1 | F:3, M:2 |
